# The Association Between Patient-Reported Outcome Measurement Scores and Preference for Specific Interventions

**DOI:** 10.1177/2374373519897761

**Published:** 2020-01-23

**Authors:** Emily Z Boersma, Joost T P Kortlever, Michael D Loeb, John McDonald, Gregg A Vagner, David Ring, Matt Driscoll

**Affiliations:** 1 Department of Surgery and Perioperative Care, Dell Medical School, The University of Texas at Austin, Austin, TX, USA; 2 Texas orthopedics, Midtown Medical II Building, Austin, TX, USA; 3 Orthopedic specialists of Austin, Austin, TX, USA; 4 Austin Regional Clinic, Austin, TX, USA

**Keywords:** clinician–patient relationship, communication, medical decision-making, patient expectations

## Abstract

To determine whether greater patient-reported symptom intensity and functional limitation influence expressed preferences for discretionary diagnostic and treatment interventions, we studied the association of patient factors and several Patient Reported Outcome Measure (PROM) scores with patient preferences for diagnostic and treatment interventions before and after the visit, a cross-sectional cohort study. One hundred and forty-three adult patients who completed several PROMs were asked their preferences for diagnostic and treatment interventions before and after a visit with an orthopedic surgeon. Patients with better physical function had fewer preferences for specific diagnostic interventions after the visit (*P* = .02), but PROM scores had no association with preferences for treatment interventions before or after the visit. A greater percentage of patients expressed the preference for no diagnostic or treatment intervention after the visit with a physician than before (diagnostic intervention; 2.1% before vs 30% after the visit; *P* ≤ .001 and treatment intervention; 2.1% before vs 17% after the visit; *P* ≤ .001). This study suggests that physician expertise may be more reassuring to people with more adaptive mind sets.

## Introduction

Surgeons often encounter patients who bring specific diagnostic or treatment preferences to an initial visit. For instance, some patients might be disappointed if an office visit does not lead to a magnetic resonance imaging (MRI) test, opioid medication, injection, or surgery. Prior studies suggest about one-third of patients arrive with a self-diagnosis prior to seeing the hand surgeon (1). This might contribute to a stronger preference for a specific diagnostic or treatment intervention. There is some evidence that a preference for intervention might reflect less effective coping strategies, which are often related to stress and distress. A recent retrospective study by Crijns et al found that greater catastrophic thinking and greater tendency to limit activity owing to pain correlate with preference for surgery. Said more simply, patients with less effective coping strategies may be more likely to choose surgery (2). Surgeons should strive to help patients make the best treatment decisions for themselves based on the best available evidence and their personal values, unhindered by misconceptions and preexisting bias (3)

Patient-reported outcomes (PROs) are increasingly used to quantify the subjective aspects of illness and to measure the impact of medical care (4). Patient Reported Outcome Measure (PROM) scores measure symptom intensity and magnitude of limitations rather than blood pressure or hemoglobin A1C.

Patients and surgeons tend to focus on symptom intensity and magnitude of limitations when making management decisions. For instance, a person with mild median neuropathy at the carpal tunnel on electrodiagnostic testing or little to no arthrosis on radiographs might be offered and accept surgery based on substantial symptoms and limitations. It is important to consider that patients with more symptoms and limitations (worse scores on PROMs) tend to have less effective coping strategies. For example, patients with less effective coping strategies may exhibit catastrophic thinking and anchor on “worst-case” scenarios (4). The pitfall here is that people with more symptoms and limitations might be more likely to have misconceptions about their illness, which places them at greater risk of making decisions inconsistent with their values. A better understanding of factors associated with expressed preferences and the change in preference for interventions before and after a visit will lead to a more value-based decision-making with better shared decision-making (SDM) strategies.

To investigate the possibility that greater symptoms and limitations might influence expressed preferences for discretionary diagnostic and treatment interventions, we studied the following: (a) What patient factors (eg, sex, age, and level of education) or PROs (eg, pain intensity) are independently associated with patient preference for (A) a diagnostic or (B) a treatment intervention, both before and after the visit with the surgeon? and (b) Are there differences in intervention preferences before and after the visit with the surgeon?

## Materials and Methods

### Study Design

After institutional review board approval (Health Science Institutional Review Board of the University of Texas at Austin; 2017-10-0096) of this prospective, cross-sectional observational cohort study, we prospectively enrolled 143 adult patients over a 3-month period in 2018. Patients were enrolled at 4 orthopedic surgery offices in a large urban area. We included all new, English-speaking patients who were aged between 18 and 89 years old visiting 1 of 11 orthopedic surgeons (5 lower extremity, 4 upper extremity, 1 trauma, and 1 spine). We excluded patients who were unable to speak and understand English. Five research assistants, who were not involved with patient care, described the study to patients before the visit with the surgeon. The first part of the survey was done before the visit and the second part after the visit with the surgeon. Completion of the surveys indicated informed consent. Ten patients declined participation because they were not interested in the study.

### Outcome Measures

Patients were asked to complete a set of questionnaires consisting of 1 questionnaire before the visit with the physician and 6 questionnaires at the end of their visit: (a) preference for diagnostic and treatment intervention before the visit; (b) demographic questionnaire consisting of age, sex, race/ethnicity, marital status, number of children, education status, work status, type of insurance, the presence of comorbidities, smoking, first visit, or second opinion; (c) preference for diagnostic and treatment intervention after the visit; (d) Pain Catastrophizing Scale short form (PCS-4); (e) 11-point ordinal measure of pain intensity; (f) Patient-Reported Outcomes Measurement Information System (PROMIS) Physical Function (PF) Computer Adaptive Test (CAT); and (g) PROMIS Depression.

### Measurements

Patients’ preferences for diagnostic and treatment interventions was assessed by letting the patients check boxes for all that applied. Diagnostic interventions consisted of X-ray, ultrasound, computed tomography (CT) scan, MRI scan, electromyography, no diagnostic intervention preference, or preference for no diagnostic intervention at all; treatment interventions consisted of physical therapy, home exercise program, injection, surgery, other intervention (medication, brace, and cast), no treatment intervention preference, or preference for no treatment intervention at all.

The PCS-4 is a validated measure of catastrophic thinking in response to nociception. It is a 4-item measure with scores rating per item from 0 “not at all” to 4 “all the time,” with total scores ranging from 0 to 16, with higher scores indicating more catastrophic thinking (defined as mis- or overinterpretation of nociception) (5). Pain intensity was measured on an 11-point ordinal scale from 0 “no pain at all” to 10 “worst pain possible” (6,7).

The PROMIS questionnaires were developed by the National Institutes of Health. The CATs are based on item response and involves a dynamic set of questions based on responses to prior questions. Computer Adaptive Tests can be completed with as few as 4 to 6 questions thereby decreasing survey burden (8). Two PROMIS questionnaires were used, PROMIS PF and the PROMIS Depression (8,9). Patient-Reported Outcomes Measurement Information System PF was used to quantify the magnitude of physical limitations, and PROMIS Depression quantifies symptoms of depression, self-reported negative mood (sadness and guilt), views of self (self-criticism and worthlessness), and social cognition (loneliness and interpersonal alienation) as well as decreased positive affect and engagement (loss of interest, meaning, and purpose) (9).

All questionnaires were administered on an encrypted tablet via secure, HIPAA-compliant electronic platform: REDCap (Research Electronic Data Capture: a secure web-based application for building and managing online surveys and databases) (10).

### Study Population

No patients were excluded from the analysis. The mean age of 143 patients was 51 ± 17 years, and 68 (48%) were men (Table 1). Mean score for PCS-4 was 4.6 ± 4.3, for pain intensity 5.1 ± 2.6, for PROMIS PF 46 ± 9.4, and for PROMIS Depression 48 ± 8.1. Sixty-nine different diagnoses were made (Supplementary Appendix 1).

**Table 1.** Patient and Clinical Characteristics.^a^

Variables	N = 143
Age, years	51 ± 17 (18-86)
Men	68 (48)
Race/ethnicity	
White	101 (71)
Latino/Hispanic	28 (20)
Others	14 (9.8)
Marital status	
Married/unmarried couple	84 (59)
Divorced/separated/widowed	22 (15)
Single	37 (26)
Level of education	
High school	30 (21)
2-year college	21 (15)
4-year college	53 (37)
Postcollege graduate degree	39 (27)
Work status	
Employed	91 (64)
Unemployed/unable to work	10 (7.0)
Retired	32 (22)
Other (student, homemaker, etc)	10 (7.0)
Insurance	
Medicare/Medicaid	28 (20)
Private	96 (67)
Other	19 (13)
Additional comorbidities^b^	
Musculoskeletal disease	43 (30)
Diabetes mellitus	10 (7.0)
Other	24 (17)
None	80 (56)
Smoking	
No	136 (95)
Yes	7 (4.9)
Visit	
First visit	132 (92)
Second opinion	11 (7.7)
PCS-4	4.6 ± 4.3 (0-16)
Pain intensity	5.1 ± 2.6 (0-10)
PROMIS Physical Function	46 ± 9.4 (24-73)
PROMIS Depression	48 ± 8.1 (34-71)

Abbreviations: PCS-4, Pain Catastrophizing Scale short form; PROMIS, Patient-Reported Outcomes Measurement Information System.

^a^ Continuous variables as mean ± standard deviation (range); discrete variables as number (%).

^b^ Multiple comorbidities possible.

### Statistical Analysis

The distributions of continuous variables and assumptions concerning normality were assessed to determine the appropriateness of the statistical tests. Continuous variables are presented as mean ± standard deviation and discrete data as proportions. We used Student *t* test to assess differences between continuous variables, McNemar test for paired observations, Fisher exact for discrete variables, and 1-way analysis of variance tests for categorical variables.

We created 4 backward stepwise regression models to identify independent factors associated with (a) the preference for diagnostic interventions (A) before and (B) after the visit and (b) the preference for treatment interventions (A) before and (B) after the visit. We included all factors *P* < .10 on bivariate analysis (Supplementary Appendix 2) in the final multivariable models (Table 2). The *C* statistic indicates the area under the curve and is a measurement for the model fit (11). We considered *P* < .05 as significant.

**Table 2.** Differences Between Intervention Preferences Before and After the Visit.^a^

Intervention Preferences^b^	Before Visit	After Visit	*P* Value	Planned/Given by Surgeon^b^
No diagnostic preferences	89 (62)	54 (38)	**<.001**	–
No diagnostic interventions	3 (2.1)	43 (30)	**<.001**	40 (28)
1 or more diagnostic preferences	51 (36)	46 (32)	.50	–
X-ray	24 (17)	14 (9.8)	.053	80 (56)
Ultrasound	2 (1.4)	1 (0.70)	1.0	1 (0.70)
CT-scan	5 (3.5)	6 (4.2)	1.0	8 (5.6)
MRI-scan	28 (20)	26 (18)	.74	19 (13)
EMG	0 (0)	4 (2.8)	.13	7 (4.9)
No treatment preferences	54 (38)	0 (0)	**<.001**	–
No treatment interventions (reassurance and guidance alone)	3 (2.1)	25 (17)	**<.001**	27 (19)
1 or more treatment preferences	86 (60)	118 (83)	**<.001**	–
Physical therapy	42 (29)	40 (28)	.75	24 (17)
Home exercise program	35 (24)	47 (33)	.07	38 (27)
Injection	26 (18)	35 (24)	.12	31 (22)
Surgery	18 (13)	16 (11)	.69	15 (10)
Other (eg, brace, cast, medication)	5 (3.5)	8 (5.6)	.55	37 (26)

Abbreviations: CT scan, computed tomography scan; EMG, electromyography; MRI scan, magnetic resonance imaging scan.

Bold values indicate *P* ≤ 0.05.

^a^ Discrete variables as number (%).

^b^ Multiple interventions possible per patient.

A priori power analysis indicated that a sample size of 136 patients would provide 80% statistical power with an α set at .05. This was based on a regression with 5 predictors if disability would account for 5% or more of the variability in preference for a diagnostic or treatment intervention, and the complete model would account for 15% of the overall variability. In order to account for 5% incomplete responses, we enrolled 143 patients.

## Results

### Preference for Diagnostic Intervention

No variables were independently associated with preference for a diagnostic intervention before the visit (Table 3). Accounting for potential interaction of variables using multivariable analysis, less preference for a diagnostic intervention after the visit was independently associated with better PF, that is, higher PROMIS PF scores (odds ratio [OR] = 0.95, 95% confidence interval [CI] = 0.91-0.99, *P* = .02; C-statistic full model = .66; Table 2).

**Table 3.** Multivariable Logistic Regression Analyses of Factors Associated With Preferences for Interventions Before and After the Visit.^a^

Dependent Variables	Retained Variables	Odds Ratio	95% CI	Standard Error	*P* Value	C Statistic^b^
Preference for diagnostic intervention before visit	Sex					0.68
	Women	Reference value				
	Men	0.52	0.24-1.1	0.20	.08	
	Level of education					
	High school	Reference value				
	2-year college	0.71	0.21-2.4	0.45	.58	
	4-year college	0.94	0.33-2.6	0.50	.91	
	Post-college graduate degree	0.42	0.13-1.3	0.25	.14	
	Smoking					
	No	Reference value				
	Yes	3.3	0.53-21	3.1	.20	
	PCS-4	1.1	0.98-1.2	0.05	.15	
Preference for treatment intervention before visit	Sex					0.62
	Women	Reference value				
	Men	0.39	0.20-0.78	0.14	**.008**	
Preference for diagnostic intervention after visit	Marital status					0.66
	Married/unmarried couple	Reference value				
	Divorced/separated/widowed	2.5	0.92-6.7	1.3	0.07	
	Single	1.7	0.74-4.1	0.76	.20	
	PROMIS Physical Function	0.95	0.91-0.99	0.02	**.02**	
Preference for treatment intervention after visit	Age	1.03	0.99-1.1	0.02	.12	0.74
	Sex					
	Women	Reference value				
	Men	0.65	0.24-1.7	0.33	.39	
	Work					
	Employed	Reference value				
	Unemployed/unable to work	No values				
	Retired	4.2	0.44-40	4.8	.21	
	Other (student, homemaker etc.)	2.4	0.26-22	2.7	.44	
	PROMIS Physical Function	0.97	0.92-1.0	0.03	.20	

Abbreviations: CI, confidence interval; PCS-4, Pain Catastrophizing Scale short form; PROMIS, Patient-Reported Outcomes Measurement Information System.

^a^ Bold indicates statistically significant difference.

^b^ The C statistic is a measure of model fit and is the area under the receiver operating characteristics curve.

### Preference for Treatment Intervention

Accounting for potential interaction of variables using multivariable analysis, preference for a treatment intervention before the visit was independently associated with gender, with less preference for men (OR = 0.39, CI = 0.20-0.78, *P* = .008; C-statistic full model = .62; Table 2). No variables were independently associated with preference for a treatment intervention after the visit (Table 2).

### Difference in Preferences

Fewer patients had no diagnostic preferences after the visit (62% before vs 38% after the visit; *P* ≤ .001), and more patients preferred no diagnostic interventions after the visit (2.1% before vs 30% after the visit; *P* ≤ .001; Table 2). None of the patients had no treatment preference after the visit (vs 38% before the visit; *P* ≤ .001), more patients had preferences for no treatment intervention after the visit (2.1% before vs 17% after the visit; *P* ≤ .001), and more patients had a specific preference for one or more treatment interventions after the visit (60% before vs 83% after the visit; *P* ≤ .001; Table 2). There was no difference in preference for a specific treatment before and after the visit.

## Discussion

Efforts to ensure that patient preferences are consistent with their values and not based on misconceptions or biases merit greater attention. A better understanding of factors associated with expressed preferences can help inform this line of investigation. This study addressed the relationship between PROM scores and patients’ preferred diagnostic and treatment interventions. In addition, we assessed the differences in preferences before and after the visit.

We acknowledge some study limitations. First, most patients were white, married, employed, and well educated (the majority had at least 4 years of college). Although enrolled in several offices and representative of the population living in the studied city (a large urban area), our results might not generalize to other populations, regions, and practice settings. Second, 100% had a specific treatment preference after the visit. An element of social desirability bias could have influenced the answers. It is unclear whether this was really their preference and if they were okay with the prescribed treatment intervention or if they gave an answer they thought their physician or family member wanted to hear. On the other hand, the answers were anonymous and private (on a tablet). Third, some patients had radiographs done before the visit which could have influenced patient’s response to the preference questions after the visit. Fourth, we measured the preference at a single point in time. There is evidence which shows that patients tend to change their preferences over time (12). Patients may not have a single preference, and people may not make purely analytical decisions. Two parallel decision-making processes are described by Tversky and Kahneman. One fast and instinctive decision process and one more deliberate and slower. The way physicians portray the relevant information may stimulate one process or another. Also, time can influence this process. After a longer period, patients can shift from the fast and instinctive decision process to the more deliberate and slower process (12).

The finding that patients with better PF had fewer preferences for specific diagnostic interventions after but not before the visit may indicate that patients with less pathology or better adaption to pathology are more likely to be satisfied with expert evaluation and advice alone. Given the evidence that as much or more of the variation in PROMs is accounted for stress, distress, and effectiveness of coping strategies compared to pathophysiology, it seems that better mental health might make people more receptive to reassurance. A little over a third of patients presented with a previsit diagnostic preference (usually radiograph or MRI), but no factors were associated with these previsit preferences. This one-third of patients with a previsit preference is in accordance with evidence found on self-diagnosis. A cross-sectional study found that one-third of patients arrived with a self-diagnosis prior to seeing the hand surgeon, and 45% had done online research prior to the visit (1). Future research might investigate whether specific cognitive coping strategies or symptoms of stress or distress are associated with greater preference for diagnostic and treatment interventions before a visit. Strong previsit preferences might indicate specific aspects of the illness.

The finding that our selected PROMs were not associated with preferences for treatment interventions before or after the visit suggests that people may, at first, be more directed and specific about looking into the cause than they are about potential treatments. These findings are consistent with our hypothesis formed based on the weight of evidence to date which suggests that symptoms’ intensity and magnitude of limitations are strongly influenced by mind-set and circumstance (2,13). In other words, PROM scores may lead to a pro-diagnostic intervention stance not because the disease is worse but because the symptoms are more bothersome, in part related to mental and social health. This is a possibility worthy of additional investigation. It may be that a maladaptive mind-set that creates more symptoms and limitations for a given pathophysiology is contributing to a greater sense that diagnostic interventions leading to a clear reason for the problem might be helpful. If this is the case, it would alter decision-making. Greater catastrophic thinking and greater tendency to limit activity owing to pain were associated with preference for surgery in a prior study (2). In further support of this line of thinking, a review of 7 studies of decision-making about spinal surgery found that severe bodily pain, poor PF, poor psychological health, and higher level of functional disability was associated with preference for spine surgery (14).

When comparing the difference in preference before and after the visit with a physician, we found that patients desire fewer diagnostic and treatment interventions after a visit with a physician than before the visit. Most patients prefer to share decisions with their physician (15). The way physicians convey the relevant medical information may stimulate a specific preference from a patient. Shared decision-making is predicated on the assumption that there is a preferred treatment and that dialogue between physician and patient will lead to the patient’s preference and decision (16). A study of scenarios of tibia plateau fracture management found that patients were influenced more by avoided losses than potential gain, emotional cues, choices reported by others, answers proposed in the question, and seemingly irrelevant options (17). Physicians’ way of explaining the diagnosis and interventions needed for this diagnosis influences the choice from the patient. For instance, Dixon et al found that using words that highlight novelty have an important influence on patient preference for robotic assisted surgery for a hypothetical diagnosis of colon cancer and that the use of more neutral language can mitigate this effect (18). We think that by guiding the patients toward a more deliberate and slower decision-making process by using this information and SDM strategies, patients will have a more deliberate and value-based decision. A study on preferences regarding SDM showed that patients also want to be guided in their decision process. Patients with Carper Tunnel Syndrome preferred to share decisions with their surgeons with a tendency of wanting more involvement in the final decision (19). Especially for those patients with more symptoms and limitations, these communication strategies are an important opportunity to become familiar with one’s values and make sure one’s decisions are consistent with those values.

## Conclusion

Lower symptom intensity and less physical limitations were associated with fewer preferences for diagnostic interventions after expert consultation. The shift toward fewer preferences for any diagnostic intervention after a visit with a physician might be a reflection of the greater ability of physician expertise to reassure people with more adaptive mind-sets—a hypothesis that merits additional study. Clinicians sometimes encounter people with substantial hope pinned on a specific intervention, but our data suggest this is the exception rather than the rule. The development of effective communication strategies might help patients of all mind-sets make decisions consistent with their values, more so with more symptoms and limitations.

## Supplemental Material

Supplemental Material, Supplementary_material_1_(1) - The Association Between Patient-Reported Outcome Measurement Scores and Preference for Specific Interventions Click here for additional data file. Supplemental Material, Supplementary_material_1_(1) for The Association Between Patient-Reported Outcome Measurement Scores and Preference for Specific Interventions by Emily Z Boersma, Joost T P Kortlever, Michael D Loeb, John McDonald, Gregg A Vagner, David Ring and Matt Driscoll in Journal of Patient Experience

Supplemental Material, Supplementary_material__2 - The Association Between Patient-Reported Outcome Measurement Scores and Preference for Specific Interventions Click here for additional data file. Supplemental Material, Supplementary_material__2 for The Association Between Patient-Reported Outcome Measurement Scores and Preference for Specific Interventions by Emily Z Boersma, Joost T P Kortlever, Michael D Loeb, John McDonald, Gregg A Vagner, David Ring and Matt Driscoll in Journal of Patient Experience
