# Supplementary material for: The Association Between Patient-Reported Outcome Measurement Scores and Preference for Specific Interventions
Source: J Patient Exp. 2020 Jan 23;7(6):1595–601. doi: 10.1177/2374373519897761 (PMC7786652; doi:10.1177/2374373519897761)
Supplement: Supplemental Material, Supplementary_material_1_(1) - The Association Between Patient-Reported Outcome Measurement Scores and Preference for Specific Interventions [file Supplementary_material_1).pdf]

Appendix 1. Diagnoses and frequencies.	
Diagnoses	Frequency
Nonspecific knee pain	9 (6.29)
Medial epicondylitis	8 (5.60)
Carpal tunnel syndrome	7 (4.90)
Finger osteoarthritis	7 (4.90)
Rotator cuff tendinopathy	7 (4.90)
Trigger finger	7 (4.90)
Adhesive capsulitis	6 (4.20)
Nonspecific shoulder pain	5 (3.50)
Biceps tendinitis	4 (2.80)
Finger fracture	4 (2.80)
Knee osteoarthritis	4 (2.80)
Acromioclavicular (AC-)joint osteoarthritis	3 (2.10)
Ankle sprain	3 (2.10)
Finger pain	3 (2.10)
Shoulder osteoarthritis	3 (2.10)
Acromioclavicular (AC-)joint injury	2 (1.40)
Distal radius fracture	2 (1.40)
Hand laceration	2 (1.40)
Hand pain	2 (1.40)
Hip labral tear	2 (1.40)
Meniscus tear	2 (1.40)
Radial head fracture	2 (1.40)
Shoulder injury	2 (1.40)
Wrist pain	2 (1.40)
Ankle fracture	1 (0.70)
Anterior cruciate ligament (ACL) tear knee	1 (0.70)
Anterior knee pain	1 (0.70)
Back pain	1 (0.70)
Bilateral carpometacarpal (CMC-)arthritis	1 (0.70)
Bilateral tibial pain	1 (0.70)
Carpal tunnel syndrome and rotator cuff tendinopathy and lateral epicondylitis	1 (0.70)
Carpal tunnel syndrome and rotator cuff tendinopathy	1 (0.70)
Carpal tunnel syndrome and trigger finger	1 (0.70)
Carpal tunnel syndrome and wrist pain	1 (0.70)
Cervical radiculopathy and shoulder pain	1 (0.70)
De Quervain tenosynovitis	1 (0.70)
Dupuytren contracture	1 (0.70)
Elbow bursitis	1 (0.70)
Elbow cellulitis	1 (0.70)
Elbow pain	1 (0.70)
Finger cyst	1 (0.70)

Finger laceration	1 (0.70)
Finger sagittal band rupture	1 (0.70)
Flexor pollicis longus (FPL) tendinosis	1 (0.70)
Hamstring tendinitis	1 (0.70)
Hip pain	1 (0.70)
Humerus fracture	1 (0.70)
Iliac lesion	1 (0.70)
Lax biceps	1 (0.70)
Medial meniscus pain	1 (0.70)
Metacarpal exostosis	1 (0.70)
Metatarsal fracture	1 (0.70)
Nonspecific hip pain	1 (0.70)
Osteoarthritis unspecified	1 (0.70)
Pelvic fracture	1 (0.70)
Pes planovalgus	1 (0.70)
Psoas tendinitis	1 (0.70)
Radial styloid tenosynovitis	1 (0.70)
Sacral fracture	1 (0.70)
Sciatica	1 (0.70)
Shoulder arthroplasty	1 (0.70)
Shoulder impingement	1 (0.70)
Sternoclavicular (SC-)joint injury	1 (0.70)
Trigger finger and de Quervain tenosynovitis	1 (0.70)
Trigger finger and ulnar nerve injury	1 (0.70)
Ulna fracture	1 (0.70)
Ulnar nerve injury	1 (0.70)
Unknown	1 (0.70)
Wrist cys	1 (0.70)

Discrete variables as number (percentage).
