# Supplementary material for: The Association Between Patient-Reported Outcome Measurement Scores and Preference for Specific Interventions
Source: J Patient Exp. 2020 Jan 23;7(6):1595–601. doi: 10.1177/2374373519897761 (PMC7786652; doi:10.1177/2374373519897761)
Supplement: Supplemental Material, Supplementary_material__2 - The Association Between Patient-Reported Outcome Measurement Scores and Preference for Specific Interventions [file Supplementary_material_2).pdf]

Appendix 2. Bivariate analyses of factors associated with preferences for diagnostic and treatment interventions before and after the visit.												
	Intervention preferences											
Variables	Diagnostic before visit	Diagnostic before visit	P value	Treatment before visit	Treatment before visit	P value	Diagnostic after visit	Diagnostic after visit	P value	Treatment after visit	Treatment after visit	P value
	No	Yes		No	Yes		No	Yes		No	Yes	
**Age**	53 ± 17	48 ± 16	0.16	49 ± 17	52 ± 16	0.33	52 ± 16	50 ± 17	0.59	42 ± 16	53 ± 16	**0.003**
**Sex**												
Women	43 (57)	32 (43)	0.08	22 (29)	53 (71)	**0.01**	49 (65)	26 (35)	0.59	9 (12)	66 (88)	0.08
Men	49 (72)	19 (28)		35 (51)	33 (49)		48 (71)	20 (29)		16 (24)	52 (76)	
**Race/Ethnicity**												
White	69 (75)	32 (63)	0.16	41 (72)	60 (70)	0.86	70 (72)	31 (67)	0.81	17 (68)	84 (71)	0.40
Latino/Hispanic	17 (18)	11 (22)		10 (18)	18 (21)		18 (19)	10 (22)		7 (28)	21 (18)	
Other	6 (6.5)	8 (16)		6 (11)	8 (9.3)		9 (9.3)	5 (11)		1 (4.0)	13 (11)	
**Marital status**												

Married/Unmarried couple	52 (56)	32 (63)		31 (54)	53 (62)		63 (65)	21 (46)		13 (52)	71 (60)	
Divorced/Separated/ Widowed	18 (20)	4 (7.8)	0.17	13 (23)	9 (10)	0.14	11 (11)	11 (24)	0.06	2 (8.0)	20 (17)	0.19
Single	22 (24)	15 (29)		13 (23)	24 (28)		23 (24)	14 (30)		10 (40)	27 (23)	
**Level of education**												
High school	15 (16)	15 (29)		12 (21)	18 (21)		22 (23)	8 (17)		8 (32)	22 (19)	
2-year college	13 (14)	8 (16)		9 (16)	12 (14)		12 (12)	9 (20)		1 (4.0)	20 (17)	
4-year college	33 (36)	20 (39)	0.08	18 (32)	35 (41)	0.69	34 (35)	19 (41)	0.46	10 (40)	43 (36)	0.24
Post-college graduate degree	31 (34)	8 (16)		18 (32)	21 (24)		29 (30)	10 (22)		6 (24)	33 (28)	
**Work status**												
Employed	58 (63)	33 (65)		37 (65)	54 (63)		62 (64)	29 (63)		23 (92)	68 (58)	
Unemployed/Unable to work	6 (6.5)	4 (7.8)		5 (8.8)	5 (5.8)		5 (5.2)	5 (11)		0 (0)	10 (8.5)	
Retired	24 (26)	8 (16)	0.23	10 (18)	22 (26)	0.62	24 (25)	8 (17)	0.45	1 (4.0)	31 (26)	**0.01**
Other (student, homemaker etc.)	4 (4.4)	6 (12)		5 (8.8)	5 (5.8)		6 (6.2)	4 (8.7)		1 (4.0)	9 (7.6)	

**Insurance**											
Medicare/Medicaid	16 (17)	12 (24)	0.63	12 (21)	16 (19)	0.38	19 (20)	9 (20)	0.89	3 (12)	25 (21)	0.40
Private	64 (70)	32 (63)		35 (61)	61 (71)		66 (68)	30 (65)		17 (68)	79 (67)	
Other	12 (13)	7 (14)		10 (18)	9 (10)		12 (12)	7 (15)		5 (20)	14 (12)	
**Additional comobordities**												
Muskuloskeletal disease												
No	65 (65)	35 (35)	0.85	41 (41)	59 (59)	0.71	70 (70)	30 (30)	0.44	18 (18)	82 (82)	1.0
Yes	27 (63)	16 (37)		16 (37)	27 (63)		27 (63)	16 (37)		7 (16)	36 (84)	
Diabetes Mellitus												
No	84 (63)	49 (37)	0.50	52 (39)	81 (61)	0.52	91 (68)	42 (32)	0.73	23 (17)	110 (82)	0.67
Yes	8 (80)	2 (20)		5 (50)	5 (50)		6 (60)	4 (40)		2 (20)	8 (80)	
Other												
No	77 (65)	42 (35)	0.82	51 (43)	68 (57)	0.12	81 (68)	38 (32)	1.0	22 (18)	97 (52)	0.77
Yes	15 (63)	9 (38)		6 (25)	18 (75)		16 (67)	8 (33)		3 (13)	21 (87)	
None												
No	38 (60)	25 (40)	0.39	21 (33)	42 (67)	0.17	40 (63)	23 (37)	0.37	9 (14)	54 (86)	0.51
Yes	54 (68)	26 (33)		36 (45)	44 (55)		57 (71)	23 (29)		16 (20)	64 (80)	
**Smoking**												

No	90 (66)	46 (34)	0.10	54 (40)	82 (60)	1.0	94 (69)	42 (31)	0.21	24 (18)	112 (82)	1.0
Yes	2 (29)	5 (71)		3 (43)	4 (57)		3 (43)	4 (57)		1 (14)	6 (86)	
**Visit**												
First visit	86 (65)	46 (35)	0.52	53 (40)	79 (60)	1.0	90 (68)	42 (32)	0.75	23 (17)	109 (83)	1.0
Second opinion	6 (55)	5 (45)		4 (36)	7 (64)		7 (64)	4 (36)		2 (18)	9 (82)	
**PCS-4**	4.0 ± 3.8	5.7 ± 4.8	**0.02**	4.7 ± 4.5	4.6 ± 4.1	0.83	4.5 ± 4.2	4.9 ± 4.4	0.57	4.6 ± 4.8	4.6 ± 4.1	1.0
**Pain intensity**	5.0 ± 2.5	5.4 ± 2.8	0.42	4.9 ± 2.6	5.3 ± 2.6	0.35	4.9 ± 2.6	5.6 ± 2.6	0.16	4.7 ± 3.1	5.2 ± 2.5	0.41
**PROMIS Physical Function**	46 ± 10	45 ± 8.1	0.42	46 ± 9.9	46 ± 9.1	0.83	47 ± 9.4	43 ± 8.6	**0.01**	50 ± 10	45 ± 9.0	**0.02**
**PROMIS Depression**	47 ± 8.3	49 ± 7.8	0.28	47 ± 9.3	48 ± 7.2	0.33	47 ± 8.2	48 ± 8.0	0.47	48 ± 8.2	47 ± 8.1	0.69

Bold indicates statistically significant difference; Continuous variables as mean ± standard deviation; Discrete variables as number (percentage); PROMIS = Patient-Reported Outcomes Measurement Information System; PCS-4 = Pain Catastrophizing Scale short form.
